# Effects of Rule Modifications on the Quality and Manner of Technical Skill Execution in Youth Volleyball

**DOI:** 10.3390/sports14040132

**Published:** 2026-03-26

**Authors:** José M. Palao, Ruth Alvarado-Ruano, Jesús Salado, Enrique Ortega-Toro

**Affiliations:** 1 Department of Health, Kinesiology and Sport Management, University of Wisconsin-Parkside, Kenosha, WI 53144, USA; palaoj@uwp.edu; 2 Department of Physical Activity and Sport, Faculty of Sport Science, University of Murcia, 30720 Murcia, Spain; r.alvaradoruano@um.es; 3 Department of Physical Activity and Sport, CEU Cardenal Spinola Centre for University Studies, 41930 Bormujos, Spain; jsalado@ceu.es

**Keywords:** team sport, development, constraints, scaling, competition

## Abstract

The aim was to assess the effect of changes in both the net height and the court size, as well as serve limitations on the manner of execution of the technical-tactical actions in youth volleyball. A total of 29 female under-14 volleyball players from three regional club teams participated in the study. A quasi-experimental design was applied. The independent variables included: (a) Tournament following the standard rules, (b) Experimental Tournament 1 (lowering of the net height from 2.10 m to 2 m, no jump serves, and a two serve per-player and -rotation maximum), and (c) Experimental Tournament 2 (lowering of net height from 2.10 m to 2 m, reduced court size from 9 × 9 m to 8 × 8 m, no jump serves, and a two serve per-player and -rotation maximum). Experimental Tournament 1 involved reductions in ball control, duration of the game phases, the occurrence of actions, and their efficacy. Experimental Tournament 2 involved increases in ball control, the duration of the game phase, the occurrence and variability of actions, and their efficacy. Lowering the net height and reducing the court size while adapting the serve rules (Experimental Tournament 2) resulted in situations that were better adapted to this population.

## 1. Introduction

Volleyball is a sport that is played by two teams on a court, which is divided by a net. Children start to play it when they are 10–12 years old or older. The reason for this late start is the motor requirements that are involved. Players cannot grab the ball, and they have to make displacements and jumps to volley or hit it. Teams can only make three ball contacts per rally, and players can only make one consecutive contact. The game is played in a small space, and there are many teammates who attempt to send the ball over the net to land in the opposite court [1]. All these aspects make it difficult for young players to control the ball. Therefore, it is common for the initial stages of player development to be focused on continuity and participation (i.e., three ball contacts by the team) [2,3]. Throughout an athlete’s development, the purpose is to develop control and quality of their actions [2]. Research studies have focused on the analysis of learning and training conditions in the early stages of the students’ and players’ development. These studies have tried to better understand how the manipulation of the task constraints influences learning and training in volleyball, especially the number of players per team, and scaling the net and court [4]. Less is known about the impact of scaling competition rules on athlete development. In the 1960s, mini-volley competition rules were developed to adapt the rules to children who start to play volleyball (under age 10, [5]). Mini-volley involves adaptations in the number of players, net height, and court dimensions, among other rules [5]. Older players (over 12 years old) play with nearly the same rules as adults, except for the net height, which is progressively higher from U-12 to U-16. However, little is known about the impact of these competition rules on game dynamics or athlete development in volleyball.

Athlete development in volleyball involves a progression in the technical techniques (e.g., types of sets or attacks) and tactical collective actions (e.g., double blocks) according to physical literacy, maturation, specialization, trainability, and intellectual, emotional, and moral development [3,6]. From a theoretical perspective (Long Term Athlete Development), various experts and coaches have proposed different approaches and progressions to develop players’ and teams’ technical-tactical skills (i.e., Volleyball Canada [3]). Scaling the sport rules is one of the proposed options to adapt the sport to the maturation and needs of the athlete. Competition rules and the game dynamics they create are the criteria used as a reference to guide training to prepare athletes for competition. However, starting at age twelve, players play with almost the same rules as adults (Table 1). One of the rules that changes is the net height, which adapts according to the sex and age group, in accordance with the anthropometric changes through maturation. The net height impacts actions carried out near the net (i.e., attack and block) as well as the time receivers have. Observational studies have shown that throughout the development process, there is decreased serve performance and increases in continuity and speed of the game, the performance of the spike and block actions, as well as an increase in the side-out performance of the team in reception [7]. However, it is not possible to establish the real impact of the maturation, training, or change in net height on this evolution in those studies. No experimental studies have been found on the impact of changing competition rules on athlete development. Most studies have focused on the impact of changes in small-sided games in training [4]. In 1998, the FIVB changed the court size (and score systems) in beach volleyball. These changes involved a reduction in the serve performance and continuity of the rallies, as well as an increase in the attack and offensive performance of the side-out team for adult male players [8]. No research was found on the impact of changes in the competition rules in developmental stages (quality and variability in the actions done).

The current paper focuses on changes to the competition rules to scale the sport for youth players. The experimental rule proposals try to increase a team’s ability to build its offense (quality and efficacy). Lowering the net height aims to facilitate the proper execution of the actions near the net, i.e., the attack and block. To avoid a possible increase in the serve efficacy that could negatively impact the ability to build the offense and the game dynamics, a reduction in the court and limitations on the serve were also tested. The reduction in the court aims to facilitate the receiver and the setter in winning the ball and building the offense. The limitations on the serve (i.e., not allowing the jump serve and limiting the number of consecutive serves done by players) have the goal to balance the serve and reception. Our study was conducted with athletes in the train-to-train stage, specifically in the phase of slow deceleration of the peak height velocity [3,6]. The goal of the proposed manipulation is to allow players to practice and apply the technical and tactical skills that prepare them to play volleyball in future stages of development. The analysis of this paper complements the analysis done in a previous research article carried out by the same research group. In that previous paper, the effect of scaling the net height, court size, and serve limitations during developmental stages [9] was analyzed from a general perspective (technical, physical, and psychological effects). Reductions in net height and court dimensions led to game dynamics more like those of older players, given the resulting balance between serve and reception. The current paper analyzes the same intervention but focuses on how manipulations of net height, court size, and serve limitations affect the techniques used, the origin, the destination, the variability, and the efficacy of the different technical-tactical actions in volleyball. This information can be useful to federations and other stakeholders in understanding the impact of different rule combinations that allow scaling volleyball to the characteristics of players. However, it is important to be aware that a short experimental study is limited because it focuses on the immediate effects of scaling on the game within a particular population and not the effect of playing and training with these rule adaptations throughout the season. Nevertheless, it can provide objective information that allows us to reflect on the impact of rule adaptations on the occurrence of different actions, techniques used by players, the manner of execution, and the quality and efficacy of the executions. The aim of this study was to assess the effects of reduced net height (from 2.10 m to 2 m) and court size (from 9 × 9 m to 8 × 8 m) on the manner of execution of technical-tactical actions in young female volleyball players (under 14). The initial hypothesis is that a modified competition will lead to greater variability in the use of technical actions, as well as improved quality and effectiveness of technical actions, especially those related to spiking and serving.

## 2. Materials and Methods

### 2.1. Participants

The sample included 29 female volleyball players from three amateur teams in the U-14 age group of a regional club competition. The players’ characteristics were the following: 13.4 ± 0.68 years of age; 1.63 ± 0.96 m of height; 55.5 ± 7.9 kg of weight; 2.85 ± 0.31 training sessions per week, lasting an average duration of 1.37 ± 0.44 h per session; and 3.21 ± 0.85 years of experience. At the beginning of the study, all players had reached puberty. Players’ guardians provided written consent after being informed of the study. The study involved three tournaments that had three different competition rules (official rules, first experimental rules option, and second experimental rules option). Over nine matches, a total of 5315 ball actions carried out by the players were analyzed. The University Ethics Committee of the research group that carried out the study approved it (ID 1944/2018).

### 2.2. Design and Variables

A quasi-experimental design was applied to evaluate the effect of modifying: the net height (from 2.10 m to 2.00 m), the serve type (no jump serves were allowed), the limitation on total consecutive serves by players (maximum of two serves per player per rotation), and the court size (from 9 × 9 m to 8 × 8 m). The game format (i.e., which set of rules was being followed) was the independent variable, and there were three conditions (Table 2): official rules (i.e., the Control Tournament) and experimental rules (i.e., Tournament 1 (T1) and Tournament 2 (T2)). The Control Tournament used the official rules for the U-14 age category, as established by the Spanish National Volleyball Federation [10].

The dependent variables included:

(a) Technical actions carried out (number of serves, receptions, sets, attacks, blocks, and defenses);
(b) Technique used to carry out the technical actions of serve (standing, jump or underhand), reception (bump or overhead), set (overhead standing, overhead jump, bump or other), attack (spike or other), block (no block, one player, or two players), and dig (bump or overhead) (adapted from Palao et al., [11]);
(c) Origin or zone of the court in which the technical action of serve (short or far-away, >1.5 m), reception (six zones of the court), set (six zones of the court), attack (six zones of the court), block (three zones of the net), and dig (six zones of the court) were done (adapted from Palao et al., [11]);
(d) Destination or zone of the court to which the ball was sent by a player in each technical action (six zones of the court) (adapted from Palao et al., [11]);
(e) Quality of the serve, reception, set, attack, block, and dig actions (on a scale from 0 to 5). The quality of the technical actions was established based on whether the technique of the players’ actions matched the key aspects of the movement (Palao et al., [11]). A quality score was assigned to each ball action according to how properly executions were done (on a scale from 0 to 5, in which 0 points meant no aspects were correctly executed, while 5 points meant that all aspects were carried out properly). The criteria analyzed for the different technical actions were: serve (ball tossing, cocking, hit surface, kinetic chain, and follow-up movement), reception/court defense/set (win the ball, orientation destination, contact surface, contact height, and kinetic chain), attack (approach and win ball, take-off, cocking, hit surface, landing), and block (win the ball, kinetic chain, arms actions, orientation, and landing);
(f) Efficacy of the serve, reception, set, attack, block, and dig actions (on a scale from 0 to 3 or 4) [12]. Efficacy was assessed according to the impact of the action on the rally [11]. On this scale, 0 was an error action, 1–3 was an action of continuity that allowed the opponent or the (own) team to play the ball (1, 2, and 3 were assigned when it did not limit the attack, it limited the attack, and it did not allow the team to attack, respectively) and 4 was a point. For reception, set, and defense (continuity actions), a scale of 0 to 3 was utilized. For serve, attack, and block (terminal actions), a scale of 0 to 4 was utilized;
(g) Team game phases (side-out complex or counter-attack complex);
(h) Efficacy of team game phases (on a scale from 0 to 4). This was established according to the effect of the action on the rally (on a scale from 0 to 4), in which 0 was an error, 1–3 was continuity that allowed the opponent or the (own) team to play the ball (1, 2, and 3 were assigned when it did not limit the attack, it limited the attack, and it did not allow the team to attack, respectively) and 4 was a point; and
(i) Duration of the team game phase (seconds). The duration of the team game phase was established from the moment that the previous action of the phase was complete until the moment that the last action of the phase was done.

### 2.3. Procedure

Data from three tournaments that were played one week after the end of the official regular season. The tournament order was the Control Tournament (CT), Tournament 1 (T1), and Tournament 2 (T2). All tournaments were held in the same conditions: indoor pavilion, atmospheric conditions, round robin competition system, order of the confrontations, and teams’ usual match routines and warm-ups. Each tournament involved three matches (two matches per team and tournament). In all the tournaments, the same procedures were used. Two fixed digital cameras (50 fps) recorded the actions carried out by the players from an elevated rear view (lateral and posterior). The actions were recorded and analyzed by one trained observer (with a Master’s in Sports Science and over two years of experience in match analysis and volleyball). The observer was trained with the observation instrument [13]. After the training period, inter- and intra-observer reliability were calculated (Cohen’s Kappa was calculated for the nominal variables, and inter-class correlation coefficient and Pearson correlation were calculated for the continuous variables). Another researcher was used as a reference to calculate the intra-observer reliability. The researcher had more than ten years of experience in sports analytics and held a sports science degree. Before and after the observation, the observer’s reliability was measured (for the nominal variables, Cohen’s Kappa; and for the continuous variables, Inter-class correlation coefficient and Pearson correlation). The minimum inter-observer reliability for the nominal variables was 0.84, and the minimum intra-observer reliability was 0.93. The minimum level of inter-observer and intra-observer reliability for the continuous variables was 0.96. The observer analyzed the matches using video. All the difficult actions and plays were reviewed with another researcher. For the intra-observer reliability analysis, 10% of rallies were randomly selected and observed to establish the reliability of the observation. The reliability level achieved an acceptable level. The difficult observations were reviewed and corrected.

### 2.4. Data Analysis

Descriptive (means, standard deviation, and percentages) and inferential statistics were calculated. Data regarding the quality of the team game phases are presented in the results in percentages. The Kolmogorov–Smirnov and Chi-square tests were used to assess the normality of the continuous and categorical variables, respectively. Data did not have a normal distribution, thus requiring the use of non-parametric tests. To assess the differences between the various tournaments regarding the continuous variables, the Wilcoxon or Mann–Whitney U tests were used. The Rank Biserial Correlation (RBC) was used to assess the magnitude of the effect size [14], utilizing the following classification [15]: minimal (RBC < 0.10), small (0.10 ≥ RBC ≤ 0.24), medium (0.25 ≥ RBC ≤ 0.39), and large effect (RBC ≥ 0.40). The Pearson Chi-square test was used to assess the difference between the various tournaments for categorical variables. Then, Cramer’s V was utilized to establish the effect size of these differences, utilizing the following classification [15]: minimal (RBC < 0.10), small (0.10 ≥ RBC ≤ 0.29), medium (0.30 ≥ RBC ≤ 0.49), and large effect (RBC ≥ 0.50). Significance was set at *p* < 0.05. The JAMOVI statistics software (version 2.4.8, Sydney, Australia) was utilized to complete the statistical analysis.

## 3. Results

With regard to the effect of the experimental rules on the actions of the serve (Table 3), in T1, significantly fewer serves were done from far distances than in T2 (*p* < 0.001, ES = 0.085), significantly more serves with short destinations were done than in both the Control Tournament (*p* < 0.05, ES = 0.106) and T2 (*p* < 0.05, ES = 0.106), significantly more proper realization of the hit surface in the serve execution was done than in the Control Tournament (*p* < 0.05, ES = 0.105), and significantly lower proper realization of ball preparation, cocking, and returning to the court after serving was done than in T2 (*p* < 0.05, ES = 0.177). There was a minimum-moderate effect size for these differences.

In T2, significantly more serves were carried out from far distances than in both the Control Tournament (*p* < 0.01, ES = 0.207) and T1 (*p* < 0.001, ES = 0.271). Significantly fewer services with short destinations were done in T2 than in T1 (*p* < 0.05, ES = 0.106). Significantly higher realization of the ball preparation, cocking, and returning to the court after serving were done in T2 than in both the Control Tournament (*p* < 0.01, ES < 0.198) and T1 (*p* < 0.05, ES < 0.178). Finally, significantly more serves that limit offense were done in T2 than in T1 (*p* < 0.05, ES = 0.132). There was a minimum-moderate effect size for these differences.

With regard to the effect of the experimental rules on the reception actions (Table 4), in T1, significantly more receptions in short zones were carried out than in both the Control Tournament (*p* < 0.01, ES = 0.098) and T2 (*p* < 0.05, ES = 0.093). Significantly less proper realization of the contact height was done than in both the Control Tournament (*p* < 0.01, ES = 0.156) and T2 (*p* < 0.05, ES = 0.270). Significantly better realization of the kinetic chain of the reception execution was done than in the Control Tournament (*p* < 0.01, ES = 0.156). Significantly less proper realization of winning the ball and contact surface of the reception execution were found than in T2 (*p* < 0.01, ES = 0.118). Significantly more receptions that did not allow the set were done than in the Control Tournament (*p* < 0.05, ES < 0.156). There was a minimum effect size for these differences.

In T2, significantly more serves were carried out with a proper realization of winning the ball, contact surface, and contact height than in both the Control Tournament (*p* < 0.05, ES = 0.156) and T1 (*p* < 0.05, ES = 0.270). Significantly, more serves with proper realization of the orientation and kinetic chain of the reception execution were done in T2 than in the Control Tournament (*p* < 0.05, ES < 0.149). Significantly fewer reception errors were made in T2 than in the Control Tournament (*p* < 0.05, ES = 0.145). Finally, significantly fewer receptions that did not allow a set were done in T2 than in both the Control Tournament (*p* < 0.05, ES = 0.145) and T1 (*p* < 0.05, ES = 0.129). There was a minimum effect size for these differences.

With regard to the effect of the experimental rules on the actions of set (Table 5), in T1, significantly fewer standing sets were done than in T2 (*p* < 0.05, ES = 0.118), significantly more sets from zone 3 than in the Control Tournament (*p* < 0.005, ES = 0.108), significantly higher proper realization of winning the ball and orientation in the set execution than in the Control Tournament (*p* < 0.05, ES < 0.105), significantly lower proper realization of the contact height in the set execution than in both the Control Tournament (*p* < 0.05, ES < 0.088) and T2 (*p* < 0.001, ES < 0.170), and significantly more sets that do not allow attack than in T2 (*p* < 0.05, ES = 0.149). There was a minimum effect size for these differences.

In T2, there were significantly higher number of standing set than in T1 (*p* < 0.001, ES = 0.118), significantly more sets from zone 3 than in the Control Tournament (*p* < 0.001, ES = 0.108), significantly higher proper realization of winning the ball, orientation and contact surface in the set execution than in the Control Tournament (*p* < 0.05, ES < 0.111), significantly higher proper realization of the contact height in the set execution than in both the Control Tournament (*p* < 0.05, ES = 0.088) and T1 (*p* < 0.001, ES < 0.170), and significantly fewer sets that do not allow attack than in T1 (*p* < 0.01, ES = 0.149). There was a minimum effect size for these differences.

Regarding the impact of the experimental rules on the attack actions (Table 6), in T1, there were significantly fewer attacks directed to short zones and more attacks directed to long zones than in T2 (*p* < 0.001, ES = 0.173), significantly higher realization of the cocking than in the Control Tournament (*p* < 0.01, ES = 0.119), significantly fewer attacks that limit opponent attacks than in the Control Tournament (*p* < 0.05, ES = 0.131), and significantly higher attacks that allow opponent attacks and fewer attacks that limit the opponent set than in T2 (*p* < 0.001, ES = 0.215). There was a minimum effect size for these differences.

In T2, there were significantly higher attacks directed to short zones and fewer attacks directed to long zones than in both the Control Tournament (*p* < 0.05, ES = 0.101) and T1 (*p* < 0.001, ES = 0.173), significantly higher realization of the cocking and incorporate of the attack execution than in the Control Tournament (*p* < 0.01, ES = 0.076), significantly higher realization of the hit surface of the attack execution than in the Control Tournament (*p* < 0.001, ES = 0.139), significantly fewer attacks that do allow opponent attacks and more attacks that limit the opponent set than in T1 (*p* < 0.001, ES < 0.215). There was a minimum effect size for these differences.

Regarding the impact of the experimental rules on the block actions (Table 7), in T1, there were significantly fewer two players block and more one player block than in T2 (*p* < 0.001, ES = 0.150), significantly fewer blocks that ended in far distances than in the Control Tournament (*p* < 0.01, ES = 0.121), significantly higher proper realization of the orientation in the block execution than in both the Control Tournament (*p* < 0.001, ES < 0.234) and T2 (*p* < 0.001, ES < 0.248), significantly lower proper realization of the landing of the block execution than in T2 (*p* < 0.01, ES = 0.111), significantly higher blocks that do not limit opponent offense than in T2 (*p* < 0.001, ES < 0.199), and significantly fewer blocks that limit the opponent offense than in both the Control Tournament (*p* < 0.001, ES = 0.224) and T2 (*p* < 0.001, ES = 0.199). There was a minimum effect size for these differences.

In T2, there were significantly more two players block and fewer one player block than in T1 (*p* < 0.001, ES = 0.150), significantly lower proper realization of the orientation in the block execution than in T1 (*p* < 0.001, ES = 0.248), significantly higher proper realization of the landing of the block execution than in both the Control Tournament (*p* < 0.001, ES = 0.144) and T2 (*p* < 0.01, ES = 0.111), significantly fewer blocks that do not limit opponent offense than in T2 (*p* < 0.001, ES = 0.199), and significantly more blocks that limit the opponent offense than in T1 (*p* < 0.001, ES = 0.199). There was a minimum effect size for these differences.

Regarding the impact of the experimental rules on the defense actions (Table 8), in T1, there were significantly higher proper realization of the orientation and kinetic chain of the defense execution than in both the Control Tournament (*p* < 0.05, ES < 0.111) and T2 (*p* < 0.05, ES < 0.119), significantly higher proper realization of winning the ball than in T2 (*p* < 0.05, ES = 0.084), significantly higher proper realization of the contact surface than in the Control Tournament (*p* < 0.05, ES = 0.079), significantly higher defenses that do not limit opponent offense than in both Control Tournament (*p* < 0.01, ES = 0.158) and T2 (*p* < 0.05, ES = 0.131), and significantly lower defense that limit opponent offense than in T2 (*p* < 0.05, ES = 0.131). There was a minimum effect size for these differences.

In T2, there were significantly lower instances of properly winning the ball, orientation and kinetic chain of the defense execution than in T1 (*p* < 0.05, ES < 0.119), significantly lower defense that do not limit opponent offense than in T1 (*p* < 0.05, ES = 0.131), and significantly higher defenses that limit opponent offense than in T1 (*p* < 0.05, ES = 0.131). There was a minimum effect size for these differences.

Regarding the impact of the experimental rules on the game phases (Table 9), in T1, there were significantly lower duration of the side-out than in T2 (*p* < 0.05, ES = 0.108), significantly lower errors in the counter-attack phase than in both the Control Tournament (*p* < 0.01, ES = 0.103) and T2 (*p* < 0.01, ES = 0.102), and significantly higher continuity in the counter-attack phase than in both the Control Tournament (*p* < 0.01, ES = 0.103) and T2 (*p* < 0.01, ES = 0.102). There was a minimum effect size for these differences.

In T2, there was a significantly higher duration of the side-out than in both the Control Tournament (*p* < 0.05, ES < 0.121) and T2 (*p* < 0.05, ES = 0.108), there were significantly more errors in the counter-attack phase than in T1 (*p* < 0.05, ES < 0.102), and there were significantly fewer actions of continuity in the counter-attack phase than in T1 (*p* < 0.05, ES < 0.102). There was a minimum effect size for these differences.

## 4. Discussion

Two experimental rules were tested to assess the changes to the technical actions and game dynamics. In Tournament 1, the net height was reduced and serve limitations were imposed without changing the court size. These changes improved some of the aspects of the serve execution (arm cocking and hit surface), reduced the serves carried out far from the serving line, and increased the serves sent to short zones (close to the net). The rules that were changed involved no changes in the different categories of the serve efficacy with regard to the standard rules. However, although the different levels of serve efficacy did not change significantly, the rule changes resulted in significantly more receptions that did not allow the opponent to build their offense in the side-out (set and attack) and reduced the actions of counterattack (blocks and defense). This involved a smaller number of sets and attacks done per player. The decrease in reception efficacy could be related to the reduction in the receptions in which the height of contact was done properly. Although there were more proper realizations of the kinetic chain, this did not occur often (19% of the receptions). The decrease in the reception efficacy impacted the way the set was executed. There was a reduction in the overhead standing set, an increase in the bump set, an increase in the sets done from zone 3, a decrease in the contact height of the ball, and an increase in the sets that do not allow an attack. However, setters won the ball and oriented themselves better than with the standard rules. The lower reception and set efficacy could be the reason for the reduction in the attack efficacy (actions that limit opponents’ actions), the increase in the attacks sent to long zones, and the reduction in the attacks sent to short zones. The lower net height allowed for an increase in the proper realization of the cocking arm action in the attacks. The way the offense was constructed impacted the block and defense. These actions had lower efficacy (more actions that did not allow them to build the offense), although players carried out better executions of the block and defense (especially court defense). This lower efficacy involved a large number of errors in counterattacks. Overall, Tournament 1 resulted in decreased efficacy of the offense and counterattack, which reduced the number of actions done by players. The changes altered the way teams build their offense (reception and set) regarding the standard rules. The game dynamics that create the rules tested in Tournament 1 differed from the evolution observed through the different developmental stages, in which players improve their ability to build the side-out [7,16]. The reduction in net height without scaling the courts accelerated the game and did not allow players to have control of the ball to build the offense. The limitations on the serve were not enough to facilitate building the offense. Tournament 1 decreased the efficacy and number of actions taken regarding the standard rules for the players. Tournament 1 did not result in changes in the variability of technical-tactical actions, except for the increase in attacks with long destination and in one-player blocks. The increase in one-player blocks was likely the result of the game acceleration.

Experimental Tournament 2 (with its accompanying lowered net height, reduced court size, and serve limitations rules) had different game dynamics than Experimental Tournament 1. There was an improvement in some of the aspects of the serve execution, and there were more serve executions far away from the serve line. The lowered net height was counter-balanced by the smaller court and the serve limitation. There were no changes in the serve efficacy regarding the Control Tournament. Scaling the court and limiting the serve resulted in increases in the quality of the execution of the reception, and the receptions in the central zone, and a reduction in reception errors. This involved an increase in the standing overhead set, the quality of set execution, and an improvement in the set efficacy (reduction in the set that does not allow the team to build the attack). This allowed players to improve the efficacy of their attacks (attacks that limit opponents), to have better quality of the attack execution, and to send their attacks to zones closer to the net. The changes in the net height and court size increased the block efficacy, the quality of the block execution, and the double blocks done by players. In defense, players make more attacks and with more efficacy in their actions (fewer defenses do not allow them to build the offense). The duration of the side-out phase increased, although there was a re-scaling of the court. With regard to the differences between the experimental tournaments, Experimental Tournament 2 resulted in an increase in counterattack errors due to more continuity in the game. The rule changes in Tournament 2 created game dynamics that allowed players to have more ball contacts, with higher quality and more efficacy. These rule changes did not increase variability, except for an increase in attacks with short destinations and two-player blocks.

The combination of scaling the court and net with the serve limitations tries to balance the actions of serve and reception, which allows players to better build the offense. The reduction in the net height allowed serve trajectories to be flatter. However, the combination of the limitation of the jump serve (usually with a higher contact point and speed) and players having less space to cover meant that the players involved were able to control the ball and organize their offensive actions. The duration of the side-out increased, which demonstrates that players had more ball control and the time to build their offense. This tendency is different from the results found in elite men’s senior teams’ results, in which the shorter the duration, the higher the chance of winning the rally [17,18]. However, it is important to consider that for elite teams, the techniques, tactics, and game dynamics are more stable and standardized. Therefore, the acceleration of the game involves an advantage in the game and an imbalance in the opponent’s actions. In the initial stages of player development, players need to control the ball and timing to carry out their actions properly and build the offense. Accelerating the game, which occurred in Experimental Tournament 1, reduces efficacy and player participation. These findings reinforce the need to analyze developmental stages using normative values for these populations.

The rule changes utilized in Experimental Tournament 2 allow players to develop game dynamics that are more similar to those observed in the older developmental stages, in which players improve their ability to develop the side-out phase [7,16,19]. These results confirm previous studies that demonstrated that a reduced court size improves the efficacy of actions in indoor volleyball training and beach volleyball competition, with the exception of the serve [8,20,21]. The results demonstrate that lowering the net height without scaling the court accelerates the game to the detriment of the players’ possibilities to control the ball. Simultaneously adapting net and court size allows them to balance their effects and, together with the serve limitation, allows for game dynamics with more control, quality, and efficacy on players’ actions. This combination of rules provided better game dynamics for the sample studied in its specific moment of development (train-to-train stage). These results may be influenced by the performance level of the sample studied here (regional level). The players of this study presented less efficacy in their actions than players who participated in the national championship, due to a large number of errors and actions that did not allow them to build the offense [16,19,22]. These rules and manipulations should be tested on different levels of the women’s competition and on men. These future studies should focus on the evolution and inter-relationship of the manipulation of different rules on the volleyball player’s developmental process.

The study of the effect of rules on game dynamics has limitations that should be considered to interpret and apply the findings of this study. This paper analyzed the immediate effects of manipulating net height, court size, and serve rules on the execution of players’ actions within a small, specific group of female players (three regional teams). Due to the fact that the findings show the immediate effect of playing with these rules in a small sample, it is not possible to establish the impact of training and competing with these rules on female player development. The order of rule implementation in the games of the tournaments (the lack of counterbalancing of the independent variable) could also impact players’ behavior due to learning or fatigue from one tournament to another. Our study focused on one stage of female players’ development (train-to-train stage); however, players need different aspects throughout their developmental stages. As previously mentioned, the level of players, maturity, and their anthropometrical characteristics influenced the results. Additional studies are needed with players of different levels (e.g., ball control), anthropometric characteristics, age groups, and sex. Nonetheless, the results show that for this sample, the current rules may not best foster young players’ participation, quality of execution, efficacy, and continuity. We used the available normative values of player evolution using the current rules implemented in volleyball as a reference to evaluate our results. The findings show that a combination of net height, court manipulation, and serve limitations allows game actions and dynamics that are closer to the following stages of development. These results show a possible way to scale and adapt indoor volleyball at the train-to-train stage for female adolescent players. The results of the analysis of the techniques used, the origin, the destination, the variability, and the efficacy of the different technical-tactical actions complement the analysis done in a previous paper by our research group [9], which focuses on the impact of the actions’ efficacy, physical actions, and psychological aspects. Future studies should analyze the effects of this combination of rules at different stages of athlete development, by sex and level of competition, and after practicing with these rules for at least a season. It is necessary to avoid viewing the sport rules for adults (e.g., Olympic Games) as the reference for youth volleyball players. More research is needed to establish the best competition rules and structure that will allow for better development of children and adolescents as players and individuals.

## Figures and Tables

**Table 1:** Progression of the rules through the different age categories in indoor volleyball.

Rules	U-10	U-12	U-14	U-16	U-18 & Senior
Number of players	4 players (no libero)	4 players (no libero)	6 players (no libero)	6 players (libero allowed)	6 players (libero allowed)
Female—Net height (m)	1.90 m	2.10 m	2.10 m	2.16 m	2.24 m
Male—Net height (m)	1.90 m	2.10 m	2.24 m	2.36 m	2.43 m
Court size (m)	6 × 6 m	6 × 6 m	9 × 9 m	9 × 9 m	9 × 9 m
Ratio of m^2^ per player	9 m^2^	9 m^2^	13.5 m^2^	13.5 m^2^	13.5 m^2^
Serve (n)	No limitations	No limitations	No limitations	No limitations	No limitations
Serve (type)	No limitations	No limitations	No limitations	No limitations	No limitations
Ball size (m)	0.62 m	0.66 m	0.66 m	0.66 m	0.66 m
Format	Best of 5 sets	Best of 5 sets	Best of 5 sets	Best of 5 sets	Best of 5 sets
Points	25 pts (5th set, 15 pts)	25 pts (5th set, 15 pts)	25 pts (5th set, 15 pts)	25 pts (5th set, 15 pts)	25 pts (5th set, 15 pts)
Score system	Rally score point system	Rally score point system	Rally score point system	Rally score point system	Rally score point system

**Table 2:** Rules implemented in the control and experimental tournaments [9].

Rule	Official Rule	Modified Rules T1	Modified Rules T2
Number of players	6 players (no libero allowed)	6 players (no libero allowed)	6 players (no libero allowed)
Net height (m)	2.10 m	2.00 m	2.00 m
Field size (m)	9 × 9 m	9 × 9 m	8 × 8 m
Ratio of m^2^ per player	13.5 m^2^	13.5 m^2^	10.6 m^2^
Serve (n)	No limitation	Max two serves per player, after that, the team rotates	Max two serves per player, after that, the team rotates
Serve (type)	No limitations	Jump serve not allowed	Jump serve not allowed
Ball size (m)	0.66 m	0.66 m	0.66 m
Format	Best of 5 sets	Best of 5 sets	Best of 5 sets
Points	25 points (5th set, 15 points)	25 points (5th set, 15 points)	25 points (5th set, 15 points)
Score system	Rally score point system	Rally score point system	Rally score point system

**Table 3:** Effect of rule changes on execution of serve in the control and experimental tournaments.

Variables	Control Tournament Net 2.10 m Court 9 × 9 m		Tournament 1 Net 2.00 m Court 9 × 9 m		Tournament 2 Net 2.00 m Court 8 × 8 m		ES (Phi/Cramer’s V)		
	n	%	n	%	n	%	CT vs. T1	CT vs. T2	T1 vs. T2
Occurrence ^a^									
Standing	189	75.3	251	94.7	261	96.7	-	-	-
Jump	54	21.5	0 ^a^	0 ^a^	0 ^a^	0 ^a^	-	-	-
Overhand serve	8	3.2	14	5.3	9	3.3	-	-	-
Origin (distance)									
Short (0–1.5 m)	240	95.6	261	98.5	233	82.6	n.s.	0.207 ***	0.271 ***
Far (<1.5 m)	11	4.4	4	1.5	47	17.4	0.085 *	0.207 ***	0.271 ***
Destination (distance)									
Short (zones 2, 3, & 4)	38	15.1	62	23.4	41	15.2	0.106 *	n.s.	0.106 *
Far (zones 1, 6, & 5)	173	68.9	162	61.1	187	69.3	0.106 *	n.s.	0.106 *
Out/Net	40	15.9	41	15.5	42	15.6	n.s.	n.s.	n.s.
Destination (laterality)									
Left	37	14.7	38	14.3	45	16.7	n.s.	n.s.	n.s.
Center	112	44.6	111	41.9	93	34.4	n.s.	n.s.	n.s.
Right	62	24.7	75	28.3	90	33.3	n.s.	n.s.	n.s.
Red/out	40	15.9	41	15.5	42	15.6	n.s.	n.s.	n.s.
Quality of execution									
Ball tossing	228	90.8	235	88.7	257	95.2	n.s.	0.086 *	0.120 **
Cocking	211	84.1	236	89.1	204	75.6	n.s.	0.106 **	0.177 ***
Hit surface	188	74.9	221	83.4	223	82.6	0.105 *	0.094 *	n.s.
Kinetic chain	144	57.4	167	63.0	157	58.1	n.s.	n.s.	n.s.
Follow-up movement	190	75.7	207	78.1	244	90.4	n.s.	0.197 ***	0.165 ***
Efficacy									
Error	41	16.3	44	16.6	43	15.9	n.s.	n.s.	n.s.
Do not op offense	24	9.6	31	11.7	36	13.3	n.s.	n.s.	n.s.
Limit offense	114	45.4	101	38.1	130	48.1	n.s.	n.s.	0.132 *
Do not allow attack	22	8.8	44	16.6	28	10.4	n.s.	n.s.	n.s.
Point	50	19.9	45	17.0	33	12.2	n.s.	n.s.	n.s.

Legend: *** *p*-value < 0.001; ** *p*-value < 0.01; * *p*-value < 0.05; n.s. = Not significant; ES (Phi/Cramer’s V): Effect Size (Phi/Cramer’s V). ^a^ The jump serve was not allowed in the experimental tournaments (T1 and T2); therefore, serve occurrences were not analyzed (per the rule-based condition of the study).

**Table 4:** Effect of rule changes on the execution of the reception in the control and experimental tournaments.

Variables	Control Tournament (CT) Net 2.10 m Court 9 × 9 m		Tournament 1 (T1) Net 2.00 m Court 9 × 9 m		Tournament 2 (T2) Net 2.00 m Court 8 × 8 m		ES (Phi/Cramer’s V)		
	n	Percentage	n	Percentage	n	Percentage	CT vs. T1	CT vs. T2	T1 vs. T2
Occurrence									
Bump	183	89.3	187	91.2	205	92.8	n.s.	n.s.	n.s.
Overhead	22	10.7	18	8.8	16	7.2	n.s.	n.s.	n.s.
Origin (distance)									
Short (zones 2 + 3 + 4)	39	19.0	56	27.3	43	19.5	0.098 *	n.s.	0.093 *
Long (zones 1 + 5 + 6)	166	81.0	149	72.7	178	80.5	0.098 *	n.s.	0.093 *
Origin (laterality)									
Left (zones 1 & 2)	33	16.1	33	16.1	47	21.3	n.s.	n.s.	n.s.
Center (zones 3 & 6)	113	55.1	103	50.2	90	40.7	n.s.	n.s.	n.s.
Right (zones 4 & 5)	59	28.8	69	33.7	84	38.0	n.s.	n.s.	n.s.
Destination									
Out/Net	2	1.0	7	3.4	3	1.4	n.s.	n.s.	n.s.
Zone 2	48	23.4	39	19.0	51	23.1	n.s.	n.s.	n.s.
Zone 3	73	35.6	93	45.4	87	39.9	n.s.	n.s.	n.s.
Others	82	40.0	66	32.2	80	36.2	n.s.	n.s.	n.s.
Quality of execution									
Win the ball	62	30.2	69	33.7	100	45.2	n.s.	0.154 **	0.118 *
Orientation destination	81	39.5	98	47.8	120	54.3	n.s.	0.148 **	n.s.
Contact surface	131	63.9	133	64.9	166	75.1	n.s.	0.122 **	0.112 *
Contact height	116	56.6	84	41.0	150	67.9	0.156 **	0.116 *	0.270 ***
Kinetic chain	17	8.3	39	19.0	35	15.8	0.156 **	0.115 *	n.s.
Efficacy									
Error	45	22.0	33	16.1	27	12.2	n.s.	0.145 *	n.s.
Do not allow set	24	11.7	45	22.0	31	14.0	0.156 *	0.145 *	0.129 *
Limit set	112	54.6	97	47.3	124	56.1	n.s.	n.s.	n.s.
Do not limit set	24	11.7	30	14.6	39	17.6	n.s.	n.s.	n.s.

Legend: *** *p*-value < 0.001; ** *p*-value < 0.01; * *p*-value < 0.05; n.s. = Not significant; ES (Phi/Cramer’s V): Effect Size (Phi/Cramer’s V).

**Table 5:** Effect of rule changes on execution of the set in the control and experimental tournaments.

Variables	Control Tournament (CT) Net 2.10 m Court 9 × 9 m		Tournament 1 (T1) Net 2.00 m Court 9 × 9 m		Tournament 2 (T2) Net 2.00 m Court 8 × 8 m		ES (Phi/Cramer’s V)		
	n	Percentage	n	Percentage	n	Percentage	CT vs. T1	CT vs. T2	T1 vs. T2
Occurrence									
Overhead (standing)	196	71.0	187	65.8	262	73.8	n.s.	n.s.	0.118 *
Overhead (jump)	6	2.2	3	1.1	2	0.6	n.s.	n.s.	n.s.
Bump	70	25.4	90	31.7	91	25.6	n.s.	n.s.	0.118 *
Others	4	1.4	4	1.4	0	0	n.s.	0.116 *	n.s.
Origin									
Zone 2	111	40.2	98	34.5	116	32.7	0.108 *	n.s.	n.s.
Zone 3	101	36.6	134	47.2	149	42.0	0.108 *	n.s.	n.s.
Others	64	23.2	52	18.3	90	25.4	0.108 *	n.s.	n.s.
Destination									
Zone 4	102	37.2	125	44.5	159	44.8	n.s.	n.s.	n.s.
Zone 3	117	42.7	105	37.4	129	36.3	n.s.	n.s.	n.s.
Zone 2	24	8.8	19	6.8	24	6.8	n.s.	n.s.	n.s.
Back zones	31	11.3	32	11.4	43	12.1	n.s.	n.s.	n.s.
Quality of execution									
Win the ball	146	52.9	181	63.7	222	62.5	0.110 **	0.097 *	n.s.
Orientation	114	41.3	139	48.9	164	46.2	0.077 *	n.s.	n.s
Contact surface	241	87.3	254	89.4	332	93.5	n.s.	0.106 **	0.074 *
Contact height	220	79.7	205	72.2	305	85.9	0.088 *	0.082 *	0.170 ***
Kinetic chain	110	39.9	132	46.5	157	44.4	0.067 *	n.s.	n.s.
Efficacy									
Error	6	2.2	9	3.2	7	2.0	n.s.	n.s.	n.s.
Do not allow attack	33	12.0	46	16.3	26	7.4	n.s.	n.s.	0.149 **
Limit attack	154	56.0	143	50.5	189	53.8	n.s.	n.s.	n.s.
Do not limit attack	82	29.8	85	30.0	129	36.8	n.s.	n.s.	n.s.

Legend: *** *p*-value < 0.001; ** *p*-value < 0.01; * *p*-value < 0.05; n.s. = Not significant; ES (Phi/Cramer’s V): Effect Size (Phi/Cramer’s V).

**Table 6:** Effect of the rules changed on the execution of the attack in the control and experimental tournaments.

Variables	Control Tournament (CT) Net 2.10 m Court 9 × 9 m		Tournament 1 (T1) Net 2.00 m Court 9 × 9 m		Tournament 2 (T2) Net 2.00 m Court 8 × 8 m		ES (Phi/Cramer’s V)		
	n	%	n	%	n	%	CT vs. T1	CT vs. T2	T1 vs. T2
Occurrence									
Spike	145	53.3	155	60.1	197	55.2	n.s.	n.s.	n.s.
Ohers	127	46.7	103	39.9	160	44.8	n.s.	n.s.	n.s.
Origin									
Zone 2	28	10.3	19	7.4	32	9.0	n.s.	n.s.	n.s.
Zone 3	121	44.5	109	42.2	138	38.7	n.s.	n.s.	n.s.
Zone 4	102	37.5	114	44.2	162	45.4	n.s.	n.s.	n.s.
Back zones	21	7.7	16	6. 2	25	7.0	n.s.	n.s.	n.s.
Destination									
Error	35	12.9	34	13.2	45	12.6	n.s.	n.s.	n.s.
Short	89	32.7	67	26.0	151	42.3	n.s.	0.101 *	173 ***
Long	148	54.4	157	60.9	161	45.1	n.s.	0.101 *	173 ***
Quality of execution									
Approach & win ball	117	43.0	110	42.6	150	42.0	n.s.	n.s.	n.s
Take-off	106	39.0	114	44.2	148	41.5	n.s.	n.s.	n.s
Cocking	130	47.8	154	59.7	198	55.5	0.119 **	0.076 *	n.s
Hit surface	209	76.8	213	82.6	312	87.4	n.s.	0.139 ***	n.s.
Landing	130	47.8	124	48.1	168	47.1	n.s.	n.s.	n.s.
Efficacy									
Error	41	15.1	40	15.5	60	16.8	n.s.	n.s.	n.s.
Allow op offense	44	16.2	51	19.8	35	9.8	n.s.	n.s.	0.215 ***
Limit set	97	35.7	63	24.4	146	40.9	0.131 *	n.s.	0.215 ***
Do not allow offense	26	9.6	36	14.0	28	7.8	n.s.	n.s.	n.s.
Point	64	23.5	68	26.4	88	24.6	n.s.	n.s.	n.s.

Legend: *** *p*-value < 0.001; ** *p*-value < 0.01; * *p*-value < 0.05; n.s. = Not significant; ES (Phi/Cramer’s V): Effect Size (Phi/Cramer’s V).

**Table 7:** Effect of rules changed on block execution in the control and experimental tournaments.

Variables	Control Tournament (CT) Net 2.10 m Court 9 × 9 m		Tournament 1 (T1) Net 2.00 m Court 9 × 9 m		Tournament 2 (T2) Net 2.00 m Court 8 × 8 m		ES (Phi/Cramer’s V)		
	n	%	n	%	n	%	CT vs. T1	CT vs. T2	T1 vs. T2
Occurrence									
One player	113	45.7	89	49.4	141	33.6	n.s.	0.121 **	0.150 ***
Two players	134	54.3	91	50.6	279	66.4	n.s.	0.121 **	0.150 ***
Origin									
Zone 2	117	47.8	80	44.4	214	51.2	n.s.	n.s.	n.s.
Zone 3	118	48.2	88	48.9	178	42.6	n.s.	n.s.	n.s.
Zone 4	10	4.1	12	6.7	26	6.2	n.s.	n.s.	n.s.
Destination									
Error	190	76.9	130	72.2	334	79.5	n.s.	n.s.	n.s.
Short	57	23.1	45	25.0	77	18.3	n.s.	n.s.	n.s.
Long	0	0	5	2.8	9	2.1	0.131 *	0.104 *	n.s.
Quality of execution									
Win the ball	45	18.2	36	20.0	72	17.1	n.s.	n.s.	n.s.
Kinetic chain	201	81.4	141	78.3	351	83.6	n.s.	n.s.	n.s.
Arms actions	65	26.3	51	28.3	106	25.2	n.s.	n.s.	0.244
Orientation	29	11.7	55	30.6	44	10.5	0.234 ***	n.s.	0.248 ***
Landing	201	81.7	151	83.9	384	91.4	n.s.	0.144 ***	0.111 **
Efficacy									
Error	12	4.9	17	9.4	28	6.7	n.s.	n.s.	n.s.
Not limit op offense	99	40.2	82	45.6	145	34.5	n.s.	n.s.	0.199 ***
Limit offense	97	39.4	37	20.6	173	41.2	0.224 ***	n.s.	0.199 ***
Do not allow offense	11	4.5	18	10.0	28	6.7	n.s.	n.s.	n.s.
Point	27	11.0	26	14.4	46	11.0	n.s.	n.s.	n.s.

Legend: *** *p*-value < 0.001; ** *p*-value < 0.01; * *p*-value < 0.05; n.s. = Not significant; ES (Phi/Cramer’s V): Effect Size (Phi/Cramer’s V).

**Table 8:** Effect of rules changed on execution of the defense in the control and experimental tournaments.

Variables	Control Tournament (CT) Net 2.10 m Court 9 × 9 m		Tournament 1 (T1) Net 2.00 m Court 9 × 9 m		Tournament 2 (T2) Net 2.00 m Court 8 × 8 m		ES (Phi/Cramer’s V)		
	n	%	n	%	n	%	CT vs. T1	CT vs. T2	T1 vs. T2
Occurrence									
Bump	196	68.1	206	74.9	260	72.4	n.s.	n.s.	n.s.
Overhead	92	31.9	69	25.1	99	27.6	n.s.	n.s.	n.s.
Origin (laterality)									
Left 4 + 5	69	24.0	72	26.2	84	23.4	n.s.	n.s.	n.s.
Center 3 + 6	122	42.4	132	48.0	168	46.8	n.s.	n.s.	n.s.
Right 1 + 2	97	33.7	71	25.8	107	29.8	n.s.	n.s.	n.s.
Origin (distance)									
Short 2 + 3 + 4	121	42.0	107	38.9	162	45.1	n.s.	n.s.	n.s.
Long 1 + 6 + 5	167	58.0	168	61.1	197	54.9	n.s.	n.s.	n.s.
Destination									
Zone 2	99	34.4	83	30.2	110	30.6	n.s.	n.s.	n.s.
Zone 3	85	29.5	101	36.7	126	35.1	n.s.	n.s.	n.s.
Others	104	36.1	91	33.1	123	34.3	n.s.	n.s.	n.s.
Quality of execution									
Win the ball	82	28.5	95	34.5	96	26.7	n.s.	n.s.	0.084 *
Orientation	97	33.7	121	44.0	134	37.3	0.106 *	n.s.	0.067 *
Contact surface	160	55.6	174	63.3	214	59.6	0.079 *	n.s.	n.s.
Contact height	125	43.4	118	42.9	170	47.4	n.s.	n.s.	n.s.
Kinetic chain	31	10.8	51	18.5	37	10.3	0.110 **	n.s.	0.118 **
Efficacy									
Error	67	23.3	42	15.3	74	20.6	n.s.	n.s.	n.s.
Do not allow set	42	14.6	64	23.3	51	14.2	0.158 **	n.s.	0.131 *
Limit set	155	53.8	133	48.4	194	54.0	n.s.	n.s.	0.131 *
Do not limit set	24	8.3	36	13.0	40	11.1	n.s.	n.s.	n.s.

Legend: ** *p*-value < 0.01; * *p*-value < 0.05; n.s. = Not significant; ES (Phi/Cramer’s V): Effect Size (Phi/Cramer’s V).

**Table 9:** Effect of rules changed on game phases in the control and experimental tournaments.

	Control Tournament (CT) Net 2.10 m Court 9 × 9 m		Tournament 1 (T1) Net 2.00 m Court 9 × 9 m		Tournament 2 (T2) Net 2.00 m Court 8 × 8 m		ES (RBC)		
Variables	X	SD	X	SD	X	SD	CT vs. T1	CT vs. T2	T1 vs. T2
Duration of complex									
Side-out complex	2.92	1.28	2.95	1.29	3.20	1.14	n.s	0.121 *	0.108 *
Counter-attack complex	3.16	1.29	3.08	1.39	3.14	1.22	n.s	n.s	n.s
	**Control Tournament (CT)**		**Tournament 1 (T1)**		**Tournament 2 (T2)**		**ES (Phi/Cramer’s V)**		
	**n**	**%**	**n**	**%**	**n**	**%**	**CT vs. T1**	**CT vs. T2**	**T1 vs. T2**
Side-out efficacy									
Error	69	33.8	74	36.1	66	29.7	n.s.	n.s.	n.s.
Continuity	103	50.5	105	51.2	117	52.7	n.s.	n.s.	n.s.
Point	32	15.7	26	12.7	39	17.6	n.s.	n.s.	n.s.
Counter-attack efficacy									
Error	115	21.3	75	13.8	143	21.7	0.103 **	n.s.	0.102 **
Continuity	325	60.2	371	68.2	407	61.7	0.103 **	n.s.	0.102 **
Point	100	18.5	98	18.0	110	16.7	n.s.	n.s.	n.s.

Legend: ** *p*-value < 0.01; * *p*-value < 0.05; n.s. = Not significant; ES (RBC): Effect Size (Rank Biserial Correlation); ES (Phi/Cramer’s V): Effect Size (Phi/Cramer’s V).

## Data Availability

The data that support the findings of this study are available from the corresponding author, E.O.-T., upon reasonable request.
